# ‘Like sugar and honey’: The embedded ethics of a larval control project in The Gambia

**DOI:** 10.1016/j.socscimed.2010.02.012

**Published:** 2010-06

**Authors:** Ann H. Kelly, David Ameh, Silas Majambere, Steve Lindsay, Margaret Pinder

**Affiliations:** a London School of Hygiene and Tropical Medicine, London, UK; b Medical Research Council Laboratories, Fajara, The Gambia; c School of Biological and Biomedical Sciences, Durham University, Durham, UK; d Ifakara Health Institute, Dar es Salaam, Tanzania; e Liverpool School of Tropical Medicine, Liverpool, UK

**Keywords:** Africa, The Gambia, Research ethics, Social technology studies, Malaria, Embedding

## Abstract

This paper describes a malaria research project in The Gambia to provoke thinking on the social value of transnational research. The Larval Control Project (LCP) investigated the efficacy of a microbial insecticide to reduce vector density and, ultimately, clinical malaria in Gambian children. The LCP’s protocol delineated a clinical surveillance scheme that involved Village Health Workers (VHWs) supported by project nurses. Combining insights from ethnographic fieldwork conducted at the Medical Research Council (MRC) Laboratories in Farafenni from 2005 to 2009, open-ended interviews with project nurses, and eight focus group discussions held with participant mothers in October 2007, we consider the social impact of the LCP’s investigative method against the backdrop of several years of research activity. We found that while participants associated the LCP with the clinical care it provided, they also regarded the collaboration between the nurses and VHWs added additional benefits. Organised around the operational functions of the trial, small-scale collaborations provided the platform from which to build local capacity. While ethical guidelines emphasise the considerations that must *be added to* experimental endeavour in southern countries (e.g. elaborating processes of informed consent, developing strategies of community engagement or providing therapeutic access to participants after the trial concludes), these findings suggest that shifting attention from supplementing ethical protocols to the everyday work of research – *embedding ethics* through scientific activity – may provide a sounder basis to reinforce the relationship between scientific rigour and social value.

## Introduction

In recent years, the scope of research ethics has widened considerably. Though the backbone of biomedical ethics continues to be informed consent – a rubric that seeks to insure respect, autonomy and privacy – it is the social value of research that currently focuses academic debate (e.g. Bhutta, 2000; Emanuel, Wendler, Killen, & Grady, 2004; Macklin, 2004). This shift in focus from individual integrity to public good reflects the need to redefine the roles and responsibilities of global scientific enterprises with regard to the welfare of diverse populations. Bioethicists have cautioned that the dramatic increase in the number of trials conducted in developing countries and the widening gap between research expenditure and health burden introduce risks to subjects that exceed procedural protocols (e.g. Benatar, Singer, & Daar, 2005). Situations of health and economic crisis introduce implicit forms of coercion into subject enrolment, placing barriers to the possibility of genuine consent and to the generation of lasting benefits (Angell, 1997; Molyneux & Geissler, 2008). Indeed, where access to clinical care is limited, the tendency of participants to associate medical research with the health care it provides is a well-documented phenomenon, often described as the ‘therapeutic misconception’ (e.g. Henderson et al., 2007; Molyneux, Peshu, & Marsh, 2004). To minimize the potential for exploitation introduced by this confusion, bioethicists argue that researchers must move beyond the individual’s health to consider “the best interests of whole populations…and the ethics of international relations” (Benatar & Fleisher, 2007: 618).

Beyond new moral vocabularies, expanding the ethical milieu requires mechanisms to elicit a public voice, and indeed, bring specific public forums into being. ‘Public consultation forums’, ‘community partnerships’, and ‘stakeholder meetings’ are now a standard feature of global research governance (Binka, 2005; Leach, Scoones, & Wynee, 2005; WHO, 2006). In western contexts, strategies of public engagement are built into policy processes, have evolved within particular institutional cultures, and articulate specific political imaginaries (Lezaun & Soneryd, 2007; Rose, 1999). But where population health is a matter of international and non-governmental intervention, these collaborations lack an administrative framework to either address or respond to the participants’ interests (Black, 2001; Cooke & Kothari, 2001; Jacobs & Shapiro, 2000). For instance, Lairumbi et al.’s study of health agenda setting in Kenya underscores the fragile links between research, policy and practice. Significantly, these authors suggest that coordinating a context-specific research agenda requires a richer conceptualisation of the range of actors who shape health care practice: “agencies that fund health reforms must strive to enlarge the decision space to accommodate local stakeholders in determining local health agendas” (2008: 745).

The purpose of this paper is to analyse the potential of investigative design to transform the settings in which experiments take place. In so do doing, we hope to reframe the operational links made between clinical care and experimental practice in terms of the positive consequences – as opposed to the ethical problems – this contextual entanglement can generate. Our example comes from The Gambia, where a Larval Control Project (LCP) took place from 2004 to 2007, under the auspices of the UK Medical Research Council (MRC). During the course of the trial, Village Health Workers (VHWs) worked alongside project nurses to conduct passive clinical surveillance of the study subjects and to provide treatment. Drawing from mothers’ opinions of the LCP articulated during focus group discussions and ethnographic material on the social transactions attendant to day-to-day research work, we consider the impact of these collaborations on the social value of research.

Our analysis of *embedded ethics* takes inspiration from recent work in science and technology studies (STS) that explores the social and material dimensions of research practice (Parker, 2007). Ethnographic studies of clinical trials describe the ways in which patients are involved in the setting of agendas and the designing of investigative protocols (e.g. Callon & Rabeharisoa, 2003; Will, 2007). The focus of these authors is less on the administrative space made for public participation or on the democratic process through which the research agendas are set. Rather, the attention here is on the way research is done – the practical alignment of actors, institutions, resources, objects and practices that underwrite the production of knowledge.

Our account of the ethical significance of the LCP’s empirical embedding of engagement is limited in a number of ways. As Lairumbi et al. suggest, promoting the social value of medical research requires the conscientious realignment of industry, academic and local health priorities. This paper, however, only considers the experimental encounter – the coordination that takes place between research subjects, fieldworkers, project nurses and village health workers around the production of data. We do not include interviews with those involved in the government health sector, nor do we draw in any depth on the perspectives of local health authorities. Our aim, then, is not to speak to the broader problems of data dissemination or the translation of research into practice. Rather, our narrow focus on the practices of the LCP is intended to regain an appreciation of the extent to which the everyday practice of research shapes its relevance.

Our discussion is organised into four sections. After a note on method, we begin with a description of the Medical Research Council’s (MRC) Laboratories in The Gambia. Understanding the institutional context of the LCP illuminates the distinct modalities of benefit associated with experimental practice in this context. We then detail the project and explain the operational advantages of integrating local practice into its protocol. The clinical impact of this collaborative framework forms the focus of discussions with mothers who enrolled their children in the LCP. Their comments and concerns allow us to reflect on the public goods generated through the practice of research. We suggest that while research projects constitute an imperfect substitute for social progress, by taking greater advantage of the collaborative potential of local actors, they can support local health systems through simple modifications in research design. Ultimately, this paper challenges the notion that medical research can – and further, should – be disentangled from the clinical contexts it investigates, maintaining that these entanglements can generate the opportunities to enhance the social value of research.

## Methods

Our data is drawn from two distinct research foci. The first was an attempt to assess the operational feasibility of a collaborative partnership between the nurses and VHWs through focus group discussions (FGDs). During October 2007, eight FGDs discussions, lasting at least an hour each, were conducted with groups of ten to fifteen women in villages of varying size and distance from the nurse stationed in the zone. About three weeks before discussions were to take place, LCP fieldworkers informed village leaders about the study and the leaders in turn informed participant mothers in their villages. Because the discussions were conducted during the rainy season, when many mothers were at work in the fields, we felt it necessary to allow all those who could to attend could the meetings, resulting in a larger than usual focus group size. Participants were asked to recount their recent illness and treatment experiences and those of their children. They were further encouraged to describe the frequency and circumstances under which mothers took children to the project nurses and the expectations they had of those clinical services. Finally, mothers were asked to reflect on the MRC’s role in Gambian society. The purpose of opening discussion to these broad concerns was to explore the extent to which their experience of the LCP might shape their perception of research and influence their decisions to participate in the future. Meetings concluded by opening discussion to any topics or concerns mothers felt had not prompted by the fieldworker or been dismissed to quickly during discussion.

Discussions were lead by AK and a senior MRC fieldworker, employed by a different trial but from the area, who acted as moderator and translator. Despite precautions taken to facilitate an open communicative exchange (Bloor, Frankland, Thomas, & Robson, 2001; Dawson, Manderson, & Tallo, 1993), the instrumental associations of this format were difficult to avoid. FGDs were conducted either at the gathering place in the village’s centre – the *bantaba* – or in open-air structures that functioned as clinics or schools, which are also used for explaining research purposes to the community and enrolling participants. The fieldworker, by his own admission, had considerable experience “motivating communities”, having been previously employed by Non-Governmental-Organisations for both health- and agriculture-focused development work. Though it yielded lively discussions, the participants’ and the fieldworker’s familiarity with the focus group format threw into question the neutrality of the responses elicited during discussions. We return to this potential bias latter in the article and, in the light of the preceding discussion, use it to illustrate our arguments as to the ethical potential of the ‘confusion’ between research and development.

FGDs were recorded and transcribed by a professional translator into the local language (Wollof, Mandinka or Fula) and then translated into English. Mothers’ views were then grouped by theme, including malaria awareness, consultation experiences, frequency of illness and views on research. Quotations presented in this paper were selected to exemplify typical or particularly illustrate comments under these themes. These quotations were also presented to the LCP staff during a project meeting. The staff’s comments provided the guide for interviews with four project nurses. The interviews, which consisted of open-ended questions, explored the nurses’ experiences of working with the VHW. The co-authors and senior fieldworker worked together on the comparative analysis of the transcripts from FGDs and interviews to respond to the project’s questions about the feasibility about the VHW-nurse collaboration and to illuminate broader themes about the value of research conducted in this setting.

This project also draws empirical insight from a long-term anthropological study on local research assistants hired by the MRC in The Gambia. Conducted by AK over a series of fieldwork visits from 2005 to 2009, this project’s methods included participant observation in daily research activities, observations of interactions between fieldwork and staff, interviews with researchers based at the MRC and district health officials, thirty narrative interviews with fieldworkers, drivers and ground staff. Thus, though our focus is on mothers’ clinical experiences within the LCP study, our interpretation of themes articulated in FGDs is supported by a broader archival and ethnographic research on the social life of clinical research in The Gambia (see Kelly, in press). In connection with a close reading of anthropological and social scientific literature on transnational trials, this empirical grounding enables a more nuanced understanding of the significance of the discussions and their outcomes.

This research was approved by the joint Gambian Government/MRC Ethics Committee, and supported by the Wellcome Trust and the National Institutes of Health (NIH Grant AI058250).

## Context

UK’s largest medical research unit in a developing country, the MRC Laboratories in The Gambia has offered a site for field and laboratory-based work in Africa since 1947. After over sixty years of location-based cutting-edge research, much of what is known about tropical disease and its control has come out of The Gambia and a large proportion of its population have participated in research. Under its current director (himself a Gambian), the MRC has fostered closer ties with the ministry of health, focusing on interventions that promise to generate effective policy recommendations.

There are other, less programmatic ways in which MRC research supports Gambian health care infrastructure. The duration of the MRC’s operations and the wide resources that come through its gates generate “collateral benefits” (King, 2000). Remote, upcountry field stations provide a point of contact for neighbouring communities, who have, over the years, become habitual hosts to research and repeated beneficiaries of clinical services provided free to participants. Moreover, even modest research projects require large numbers of staff to recruit participants, collect data, drive the cars, clean the labs, and prepare meals for the staff. These jobs provide a major source of employment for Gambians, second only to agriculture.

However, despite the enduring presence of its buildings, the boundaries between Gambian society and the institutional capacities of the MRC remain sharply drawn. Both therapeutic attention and employment is intermittent. Furthermore, it is MRC policy not to hire anyone if they have recently worked for the state. A waiting period of six months before contracting staff is meant to prevent direct competition with government hospitals. In the main, MRC employees are hired for a particular research project; it is therefore, not unusual to come across fieldworkers who have worked under the auspices of the MRC for twenty years or more, but only on short-term contracts. While it provides the critical infrastructure for the outsourcing of trials into the Gambia, the MRC is not a clinic, nor do the innovations it trials produce speak to the interests of the state (Cassidy & Leach, 2009).

Set within the institutional parameters of the MRC, research projects thus encompass two distinct modalities of benefit. The first, associated with work within the laboratory compound, yields *potential* advances (publications, research grants, innovative therapeutics) that are at once far-reaching and far-off. Research generates a *continuous* flow of information, which processed through ordered protocols and standardized spaces and is effectively placeless. The second, *contiguous* with the Gambian people and landscape, provides immediate enhancements to Gambian wellbeing (health care, bed-nets, jobs, a centre for community activity) but is constrained to the resources of a singular research budget.

Like other trials before it, the Larval Control Project (LCP), was lodged between these potential and immediate axes of progress. However, as an experiment in public health management, its protocol emphasised the *sustainability* of the method on trial. In the following section, we describe the connections the LCP forged from experiment to future governmental intervention. Here we analyse the operational value of ‘embedding’ and track the intersection of ethics and method.

## The larval control project (LCP)

The Larval Control Project (LCP) was a massive undertaking. Over the course of two years, a microbial larvicide (*Bti*) was applied across four zones along the north and south banks of the Gambia River, each approximately 100 km^2^. Larval habitats are generally associated with human activity; typically they can be found in almost any body of still water. Because breeding grounds are often transient and unpredictable, larval control programs require exhaustive and continual surveys of the intervention area. With villages located between one and eight kilometres from the river, the study areas encompassed a wide range of micro-climates from sedge to grassland, rice fields to mangrove forest. *Bti* is safe for non-target organisms, and because it contains multiple toxins its use is highly unlikely to result in resistance. The downside of larviciding in an area with extremely dynamic aquatic habitats is that it must be re-applied on a weekly basis. Equipped with heavy spray packs or buckets, larvae dippers, and detailed maps, teams of three to four applicators walked abreast, roughly eight metres apart, across transepts of two kilometres in length and one hundred metres in width several times a week (Majambere, Lindsay, Green, Balla, & Ulrike, 2007).

Transforming the Gambian landscape into an object of entomological management took a degree of meticulous attention and physical stamina that could only be achieved through a large-scale collaborative effort. Though we do not have room to describe the training of spray teams in detail, it involved no less than sixty local Gambians to routinely locate, record and treat all potential breeding grounds from floodplain to brick-pit. This experimental set was designed not only to demonstrate the efficacy of *Bti*, but also to produce knowledge about a specific *policy*; it was, in a sense, a pilot study for incipient government programs. What was on trial, then, was a community-based system of management: could the training of local spray men undergird an effective, state-led eradication process? As a model for future public health practice, the value of the LCP was thus understood not only in entomological terms. In addition to establishing the larvicidal action of the intervention in the field, the project aimed to demonstrate the *clinical* effectiveness of a locally coordinated method of mosquito surveillance in reducing the incidence of malaria. This objective added another layer of logistical complexity between experimental protocol and fieldwork. First, the LCP needed to recruit a human population, in this case, two thousand children – five hundred in each zone – aged six months to ten years. This initiative required lengthy discussions with village leaders and meetings with community members many of whom were highly sceptical of MRC motives.

Second, to monitor the impact of such a continuous and incremental intervention necessitated a measure of disease incidence – the number of malarial attacks in a set population over a given time. Thus in addition to a bi-annual collection of blood samples, fieldworkers and nurses were stationed in villages during the rainy season, to record cases of malaria, survey those who had travelled, and provide onsite care at any hour. Adequate coverage relied on the strategic placement of clinical staff and close collaboration with the VHWs. Two nurses and one field assistant were stationed in key villages in each of the LCP four zones for the duration of the rainy season (May–November). During the day, nurses waited for patients from the neighbouring homes, and in the afternoon made visits to the farthest villages within their catchment area. If a study participant was considered too ill to treat onsite, he or she was assisted with transport to the hospital.

Village Health Workers (VHWs) were critical figures in this surveillance strategy. Gambia’s health care system is based on a three-tier system of referral, with local primary health care villages at its base, rural clinics operating at the district level and three national hospitals at its apex. Village development committees – a grassroots institutional tier intended to encourage rural communities to become more proactive in the development process – select village health workers, who are given six weeks training in preventative and curative medicine (Davis, Hulme, & Woodhouse, 1994). Occasionally, VHWs receive payment from research projects to serve as reporters, informing MRC staff of cases occurring in their villages that might be relevant for specific investigative purposes. In a similar capacity, they provide a contact point through which agencies can mount programs in the village.

The role ascribed to the VHW in the LCP protocol surpassed that of community-liaison. If larval controls were to prove effective as a public health intervention, the LCP had to be integrated within the existing health system. The LCP protocol described a close partnership between VHWs and nurses: while the latter provided diagnostic support and pharmaceuticals, the former were responsible for treatment. Close collaboration with local Gambians in the day-to-day operation of the LCP had clear advantages. As with larval control, local knowledge bolstered the empirical capacity of research. The village health workers’ familiarity with their communities – like the spray-men’s knowledge of the flood plains – meant that they were alert to the health of the participants. These modes of participatory practice yoked technologies of disease assessment to site-specific techniques of disease control.

Though the efficacy of community health workers in the detection of malaria has been demonstrated in other settings, here the VHW clinical skills were found wanting (e.g. Lapau, 1983; Ruebush, Weller, & Klein, 1994). Functioning as a panacea for a weak and underfunded health system, VHWs are not provided with adequate drug supply or continuing support (Menon, 1991). Few were able to read and write, and fewer still had any formal education. Beyond the six weeks of training the VHW workers received following their selection, the VHW handbook, which had not been updated since its release in 1980, is the only formal instrument of clinical support they’re given – and copies of that text are almost impossible to find. Moreover, as opposed to the Traditional Birth Assistants (TBAs) who historically occupied a role in the villages as healers, the VHWs introduced a new social actor into the local political ecology (Cham, MacCormack, Touray, & Baldeh, 1987). During the study, VHWs were positioned between government, community and, critically, the MRC, and thus were enmeshed in sensitive intra-village negotiations. Rather than facilitating community access, these political entanglements entrenched distrust of research, leading to high dropout rates.

Though it posed clear challenges, involving the VHWs was an investigative priority. Thus, at the start of the rains, LCP staff ran a series of workshops in conjunction with the relevant district health teams to retrain VHWs to recognise the signs of malaria and anaemia and to treat these appropriately. Treatment sheets and prescription slips that used pictorial representation (e.g. suns for *chloroquine* and stars for *fansidar*) proved invaluable tools towards the accurate delivery of medicine at appropriate doses. The PI devised a three-part treatment strategy, whereby participant mothers were asked to approach project nurses when their children fell ill. Following diagnosis, the nurse would issue the mother a pictorial prescription slip to deliver to the VHW. The VHW, who, at the start of the trial had been issued tins of anti-malarial drugs and antibiotics, matched the prescription sheet to the labels on the containers.

Entering the LCP’s second year, the success of this partnership was uncertain. Did the VHWs’ involvement in therapeutic delivery impact mothers’ decisions to seek care from the nurses? How, moreover, were the different roles of the nurses and VHW understood? With these questions in mind, we believed that a series of focus groups and interviews with the mothers of participant children might provide critical insight. In addition to revealing any programmatic errors in case detection, we thought the discussion would allow us to forestall any misunderstandings about the benefits the mothers might expect from the research team in the future. But rather than clarifying the investigative character of the LCP, these discussions revealed how the project’s significance was determined, to a large degree, by the institutional workings of the MRC.

## “We see them together”

Comments made during discussion groups clearly indicated that the nurse-VHW collaboration had been an effective way both to monitor malaria cases and to treat them. Mothers were aware of the nurses’ presence and purpose in the villages, and felt comfortable approaching them in the event of illness. More broadly, their experiences of the trial had been largely positive. Many mothers expressed gratitude to the LCP staff for the reliable, accessible and consistent care they and their families had received: qualities they attributed not only to the nurses’ training and their supply of drugs but also to the close relationship they fostered with the VHWs. Rather than functioning as gatekeepers, the VHW were enrolled in the clinical situation as partners. The leitmotif of discussions was the coordinated approach to treatment and diagnosis. In the words of one participant, “we seek health care from Saja [nurse] and Kebba [VHW] because we know they work hand in glove…Saja and Keba are twins; they meet and greet us together, they diagnose together”. This service represented a substantial improvement in theirs and their children’s wellbeing. Whether the nurse was stationed in the village or not, participants felt that the clinical services provided by the LCP were readily available and represented a vast improvement in the health of the village.

“Like sugar and honey,” another suggested, “that is how we see them in our community”. The clinical character of that collaboration depended on whether or not nurses were stationed in a village; when they were, VHWs and nurses were often seen simultaneously, and thus, treatment and diagnosis were experienced as integrated. For participants who lived at a distance from the LCP’s key villages, the roles were more polarized: VHWs were more directly associated with treatment, and nurses with diagnosis and clinical advice. However, in either case, participants felt equally strong that project nurses and VHW reinforced each other. Those who had, at one time or another, experienced difficulty in locating the nurse, reported that by working in tandem, the VHW acquired some of the nurse’s clinical capacity. As one participant from a village 16 km from the nearest government clinic commented, “if Fama [the nurse] is not around, Abdoulie [the VHW] could come because he has the experience of administering drugs and, in this way, the nurse is staying with us permanently”.

Nurses shared the mothers’ view that the VHWs played a central role in providing adequate clinical coverage, particularly during the rainy season, when illness spiked. They also suggested that collaborations with VHWs were invaluable in maintaining good relations with the communities. “We teach them about diagnosis and they tell us about the illnesses in the community,” said one nurse responsible for one of the more remote areas, “he [the VHW] has been very influential in encouraging participants to come to the clinic and directing them on how to take their medicines”. They stressed the responsibilities placed on the VHWs, for which they found many unprepared, particularly where VHWs were appointed for political reasons. At the very least, they felt that working with the LCP team had imbued the VHWs with a greater degree of professionalism: “A good health worker will not travel and is ready to stay with the community…but with the LCP support, they see this as something they can do”.

Arguably, the VHW and the nurse would not have made such a significant impact, if the clinical care on offer remained limited to the project. Nurses advised VHWs on all aspects of health practice, even with child-delivery, a role traditionally assigned to traditional birth assistants (TBAs). Though hesitant at the start, according to the nurses, community members became comfortable consulting nurses for more general health problems. Though the time they spent in the villages helped, the nurses believed that level of trust was a direct result of the close relationships they developed with VHW, “living in the house with the VHW, sharing meals, being there like brothers”. That fraternity not only served to tighten the connections between the project and the village, but in some cases, between the community and VHW, transforming a position allocated on the basis of intra-village connections – and often antagonistic – into one chiefly defined by therapeutic care. “The nurse has carried Pateh [VHW] a great deal, with medicines and knowledge…together they help us with the difficulties we face”.

Finally, it seemed that those relationships inflected the community’s perception of the MRC. While, as in the case of other MRC projects described elsewhere, some mothers had heard the rumours of the MRCs intention to steal their blood (e.g. Fairhead, Leach, & Small, 2006; Geissler, Kelly, Pool, & Imokhuede, 2008) the LCP had dispelled these fears. “Anybody who comes here and says he is from MRC will be accepted with 10 hands” one woman said, “I am very happy with MRC for the work they are doing, since the project started we and our children are free from difficulties.”

## Embedding and mobilisation

Concurrent with these notes of satisfaction and appreciation, the participants sounded concerns about the limited duration of the LCP, its status as an MRC *project*:

You know that the project finishes and we will suffer…you will be leaving us and we will go back to difficulties, travelling to get treatment, paying for medication, a VHW having no supply.

While medicine is subsidised for children under ten by the Gambian government, and free for children under five, the mothers emphasised the difference that free, proximate, quality care had made to their lives. But though mothers expressed dismay at the LCP’s closure, they did so almost as a matter of course. Across the groups, mothers waved away the topic of the LCP closure as somehow too obvious for discussion: “why do you ask…you know what will happen”’; “we know this will end”; “even you yourself know that”. One mother joked that they would have no problems when the LCP concluded, because she had found someone in the village the nurse would find too irresistible not to marry.

Whatever ‘misconception’ the mothers’ had about the LCP’s investigative aims, they seemed clear as to the inevitability of its closure. Further, their expectations of the LCP were formulated through reflections on the MRC, their experiences with the institution and their understanding of its function with regards to The Gambia. In general, the MRC was described as a “good” institution that is here to “help” Gambian communities, but that nonetheless had its own motives: “MRC takes knowledge back to the UK”; “working with us the MRC also gets benefits.” When pressed to differentiate between the MRC and a government hospital they did so not only in terms of the free and quality care the former offered but also the time-bound nature of its provision:

The only difference is in the medical bills, the fares we pay to get there, and that the MRC tests for many things before giving my child treatment…but this care will end, all projects have a time to end.

Thus, while participants tended to conflate research and treatment, they emphasised the conditions under which the two intersected. Their requests were not for a *continuation of* treatment, but rather for *repeated opportunities* for enrolment: “our message to the MRC is give us more future projects; there is much happiness and gratitude for their participation…we hope they will come again and again”.

It would be inappropriate to read these mothers’ willingness to engage in more research as an aspiration for innovation. Their understanding of the experiment as a distributor of short-term aid points to the ways in which research in The Gambia is institutionalised in the public health system. In their investigation of mothers’ engagement with a Pneumococcal Vaccine Trial, Fairhead, Leach and Small (2006) examine the logic through which mothers decide to enrol their children in research and, further, describe the precarious nature of that choice. ‘Being with the MRC’ entitles participants to free medications, but also renders them vulnerable to blood-theft. Rather than evidence of a failure to understand medical practices, or alternatively, an articulation of the occult, rumours of blood-stealing are here understood to be reflective of the different economies involved in the production of knowledge: “Joining,” they argue, “involves transactions” (2006: 1117); the giving of blood samples and the receiving of therapeutic care is inextricably embedded in the power imbalances and inequalities attendant to global medical sciences and the bioscience industry.

That transactional logic draws attention to the potentials risks of the LCP’s embedded engagements. As we argued above, involving the VHWs had decisive operational advantages – for the most part, their familiarity with the community enhanced clinical surveillance. But one could also easily argue that these relationships were not only good for data-capture but for sample-size. In an interview, the senior fieldworker noted the remarkable levels of enrolment and retention the LCP achieved even in communities he knew to be hostile to research. He attributed this success to the time and energy the research team had committed to working with people ‘on the ground’: “after our work with these people, they are ready for us, for any project, and at anytime”.

This imperative to pave the way for future study casts doubt on the genuine character of mothers’ responses. Mothers might be more inclined to put a positive spin on the LCP’s practices if they believe this attitude would encourage the MRC to continue to conduct studies in their area. But more troubling than the challenges this outlook brings to our assessment, is that it reflects the instrument potential of close-knit relationships between research staff and the community. In addition to a conflation between clinical care and experimental protocol, the mothers’ enthusiasm intimated the lack of distinction between ‘capacity-building’ and ‘research capacity’. In light of the mothers’ commitment to enrolling ‘anytime’, one might argue that rather than making substantial improvements to the conditions of health provision, the embedding of research serves primarily to render local conditions more amenable for investigation. For instance, might not involving the VHWs work to enforce adherence to research protocol and thus, transform hostile communities into compliant ones?

The considerable overlap between the format of the FGDs and that of participant recruitment brought these issues to the fore. During meetings, the fieldworker would occasionally abandon his inquisitive indirection for a more didactic tone. One mother’s story about her child’s recent fever became an opportunity to remind the group how to recognise malaria symptoms. When another described her father’s recent illness, the fieldworker suggested that the mother should bring him to the project, even though this was technically out of the nurse’s remit. Interestingly, when the fieldworker stepped out of the role of impartial facilitator he did not become a mouthpiece for the MRC, dwelling on the responsibilities of trial participation or attempting to dispel rumours of blood-stealing when they were mentioned. What he did emphasise was the importance of group cohesion, whether it was for health checks or for communities meetings, like the one we were conducting. Under his direction, FGDs were not merely an instrument of knowledge but a critical assembling device, a form of collective action. Driving back, from one of the villages in Zone 2 he described his concern that its inhabitants had become ‘too individualistic’: “people here do not yet understand how to take responsibility for the health of their community…We must teach them to act together if things will ever improve.”

Like the spray men in the fields or the VHWs working with the nurses, these FGDs blurred the line between research and development. These modes of community engagement entail distinct temporalities, succinctly captured by the phrase “ready for us anytime”. The transience of ‘anytime’ is replicated on a larger scale: The Gambia’s attractiveness to academic and industry-led research brings with it little by way of investment, providing only temporary infrastructures for social and economic development. But though the LCP was subject to the same temporal limitations attendant to any ‘project’, as a source and a site for training there were residual effects. The LCP trained VHWs in malaria diagnosis and treatment and local residents in larval recognition and disease control. At the trial’s closure a large shipment of DDT made its way to The Gambia. The presence of a group of local residents trained in environmental management advanced a nation-wide indoor residual spray program. Embedded in the health system, it aligned the techniques to control transmission with the local partners who could become responsible for their implementation.

During a discussion in a remote village on the south bank of the river, a mother described how her and her baby’s life had been saved when the nurse intervened during a complicated home delivery. She concluded her story with a request to equip the VHW with the capacity to help with child-birth in the future: “here, women lose a lot of blood during delivery and we are too weak to stand this…we are asking MRC when they go to support Abdoulie, equip him with the instruments and knowledge, to help us with this problem.” For mothers, the collaboration between the VHW and the nurse meant that the benefits of research were not wholly contingent on experimental practice. The trial led to improvements in health and in practice, simply by teaching VHWs. In the words of one mother: “now, there are two-way benefits because the VHW receives education and then he tells us about the disease, MRC learns from us and takes this knowledge back to UK”.

It was only a beginning; mothers worried about the VHWs’ capacities absence the material support and guidance provided during the trial. But embedding the LCP into the Gambian landscape had set in motion practical enhancements, an exchange of knowledge that linked investigative practice and to improvement in health provision. In addition to providing ‘future projects’ mothers articulated hopes for a different commitment: “We are praying for the MRC not to leave but when they do leave let them continue to empower the village health worker, teach him and supply him with enough medication”.

## Conclusion: the end(s) of research

Among low-income populations, both in developing and developed world, social scientists have documented cases of patients who strategically negotiate experimental settings for therapeutic access (Biehl, 2007; Petryna, 2009; Timmermans & McKay, 2009) or where compensation is offered, enrol in trials as a form of work (Rajan, 2002). These pragmatic tactics run counter to the altruistic volunteerism that is meant to characterise the motivation to participate in research. Like our study of the LCP, these accounts point to how deeply research is entangled in systems of public health. As international regulatory bodies take into account that reality, the ethical dimensions of research protocols are increasingly assessed in terms of their investment in local public health or scientific capacity. Despite proclamations that “the whole research endeavour should be created as a partnership” (Nuffield Council on Bioethics, 2005: 60), exactly how those partnerships will be drawn and to what end, remains unclear.

This paper has considered one example of research partnership, that between local health personnel and research employees. The social value of organising collaborations around everyday research activity is clearly not of the same order as that associated with using the input of national health ministries to set research agendas. However, the mundane effort to integrate experimental method with local practice constitutes a significant step in squaring the interests of a northern-dominated “scientific worldview” – medical innovation – with the priorities of those who enrol in research – to alleviate suffering (e.g. Benatar & Fleischer, 2007). In other words, it relocates the advancement of knowledge to something that develops through the process of research, not a product delivered at its conclusion. Rendering upcountry Gambia amenable to surveillance required an alliance between civic and scientific epistemologies; reconfigured by experimental *space*, upcountry Gambia became a *place* of public health management. LCP practices transformed villages and flood plains into venues of knowledge – not merely by bounding and designating its features for scientific investigation, but by generating a local community who might do so in the future.

In light of the WHO’s (2006) emphasis of the importance of human resources in ensuring population health, there has been renewed interest to revisit the feasibility of VHW programs. A recent review concluded that to impact health outcomes significantly, VHWs had to be carefully selected, provided with appropriate training and continuously supported (Anand & Bärnighausen, 2004). Thus, for many low-income countries, effective VHW systems are not realistic options. However, if the training of VHWs and TBAs became a requirement for all research – like informed consent – clinical projects could potentially make those programs more sustainable. Moreover, it has been shown that VHW programs are most effective where communities are actively involved in their planning and implementation (e.g. Gilson et al., 1989). Extending the techniques of community engagement – now a fixture in transnational research practice – to facilitate the democratic oversight of local health care initiatives could serve to strengthen relations between VHW and their community.

There are risks in fostering those connections. On one hand, there is the problem of coercion: as the focus group discussions illustrate, close partnerships between communities and staff can disrupt the boundary between communication and mobilisation, introduce social pressures to enrol, and ultimately, reduce the democratic potential of these engagements. On the other, relying on informal providers to support VHWs beyond project time-lines, introduces issues of sustainability (Street, 2009). Indeed, while the MRC might be a good example of the kind of research institution capable of taking on that responsibility, the recent decision to shift funding from the Gambia to other countries in the regions, suggest that even here the priorities of international research and community health needs are not easy to square.

However, the reality for the participants in the LCP, as for many in low-income settings, is that research is not experienced on a project-to-project basis (Gikonyo, Benjon, Marsh, & Molyneux, 2008). The benefits single research projects could offer by involving local practitioners in running the research, we would argue, are potentially significant. The argument is that the participatory potential of the LCP’s investigative strategy is unique to public health research, where what is at stake in situating experimental work is the production of stable policy. A further point might be made that this strategic design belongs particularly to malaria interventions that have historically deployed local-management and community-based methods of control (Panter-Brick, Clarke, Lomas, Pinder, & Lindsay, 2006). But regardless of investigative focus, all research takes place in *a place*. It also requires local people to collect data and enrol participants. This mundane work entails obviously knowledge-transfer, opportunities and provides the basis for a more equitable partnership than the exchange of participation for project-based health care.

The challenge is how to ensure that the embedding of engagement is directed towards improving local health infrastructure rather than simply boosting research capacity. In the past, medical research ethics has sought to protect the interests of research subjects by reinforcing the distinction between the activities of research and the promise of therapeutic benefits through informed consent guidelines. While informed consent remains a concern, ethical debate has shifted focus; ethicists now demand that researchers find ways to augment their impact in local health care settings (Benatar, 2004). The thrust of this argument is to widen of ethical discussion from the relations between researchers and participants to that between global institutions and nations. However, we would argue that in addition to changes in research policy and regulation, day-to-day investigative work can provide a powerful means of promoting social value. How to develop and extend these methods is, to our minds, the most pressing question bioethics faces today.
